# Gang membership, firearm victimization, and mental health in a national sample of U.S. adults

**DOI:** 10.1186/s40621-026-00656-7

**Published:** 2026-01-24

**Authors:** James A. Densley, David C. Pyrooz, Jillian K. Peterson

**Affiliations:** 1 School of Criminology and Criminal Justice, Metropolitan State University, Saint Paul, MN 55014 USA; 2 Department of Sociology and Institute of Behavioral Science, University of Colorado Boulder, Boulder, CO 80309 USA; 3 Violence Prevention Project Research Center, Hamline University, Saint Paul, MN 55106 USA

**Keywords:** Gangs, Firearms, Victimization, Mental health, Violence

## Abstract

**Background:**

Firearm violence in the United States is highly concentrated within specific demographic, economic, geographic, and social population groups. Prior research indicates elevated violence exposure among gang-involved individuals, but the extent and mental health implications of firearm victimization at the national level remain poorly understood.

**Methods:**

We analyzed data from a national online survey of 10,000 U.S. adults fielded in 2024. Respondents self-reported lifetime gang membership and lifetime exposure to five forms of firearm victimization: presence at a mass shooting, gun threats, being shot at without injury, accidental gunshot injury, and intentional gunshot injury. Weighted descriptive statistics compared prevalence by lifetime gang status. Multivariable logistic regression estimated adjusted odds ratios controlling for several covariates. Among victims of firearm violence, self-reported psychological impacts, including anxiety, fear, depression, panic attacks, and post-traumatic stress symptoms, were assessed.

**Results:**

People with a history of gang involvement reported substantially higher lifetime exposure to all forms of firearm victimization. After adjustment, gang involvement was associated with 2–4 times greater odds of firearm exposure across outcomes. Psychological impacts following firearm victimization were prevalent in both gang and non-gang groups. Differences in reported mental health impacts by gang status were generally modest, with relatively few statistically significant differences in adjusted models.

**Conclusions:**

Firearm victimization is highly concentrated among people with a history of gang involvement, and such exposure is associated with substantial psychological distress. Mental health impacts were broadly similar across gang-involved and non-gang victims. These findings highlight the importance of recognizing gang-involved adults as a population with disproportionate exposure to firearm violence and significant trauma-related needs within a public health framework.

## Background

Firearm injury is a leading cause of death in the United States and remains highly concentrated within specific populations and places [1, 2]. Although national mortality statistics highlight elevated firearm suicide rates in rural areas and high homicide rates in urban centers [3], these aggregate patterns obscure substantial inequalities in nonfatal firearm exposure. Research consistently shows that firearm violence is clustered within a small share of neighborhoods and social networks, producing repeated exposure for some individuals while leaving others largely untouched [4]. Black and Indigenous adults report substantially higher lifetime exposure to nonfatal shootings than White adults, even after accounting for socioeconomic differences [5], and individuals released from juvenile detention experience firearm mortality rates many times higher than their peers [6]. These disparities reflect intersecting structural disadvantages—including concentrated poverty, housing instability, racism, and labor market exclusion—as well as the cumulative effects of prior violence exposure [7–9] and network proximity to others at high risk [10, 11].

Street gangs sit at the intersection of these structural and relational processes. Gangs are commonly defined as durable, street- or prison-based groups who share a collective identity and are involved in illegal activity [12, 13]. Decades of research demonstrate that involvement is associated with elevated risks of both violent offending and violent victimization, even after accounting for individual characteristics and neighborhood context [14–17]. Group processes (e.g., retaliatory norms, rivalries, and collective identity) generate risks that extend beyond individual behavior [18–21]. As a result, gang-involved individuals are disproportionately embedded in social networks where violence is both more likely and more consequential [22]. Prior research also documents elevated levels of traumatic stress, depression, and post-traumatic stress symptoms among gang-involved youth and adults [23–27], including relative to other violent or justice-involved populations [28–30].

Despite this extensive literature, several gaps remain. First, national victimization surveys rarely include measures of gang involvement, limiting understanding of how firearm exposure is distributed across gang-involved and non-gang adults in the general population [31]. Studies of “mass shootings,” for example, often exclude incidents labeled as “gang-related” [32], potentially omitting a population for whom firearm violence exposure is routine rather than exceptional. Second, gang research has largely relied on localized or institutional samples, making it difficult to assess whether observed patterns generalize nationally [33]. Gang membership is relatively rare in the general population [34, 35], but gang-involved individuals bear a disproportionate burden of serious violence [36]. Finally, although a growing body of public health research documents the psychological consequences of firearm violence [37–41], relatively little work has examined whether the mental health impacts of firearm victimization differ across population groups, including for individuals with a history of gang involvement.

Research on repeated exposure to violence offers competing perspectives relevant to this question. A disadvantage saturation hypothesis posits that individuals facing multiple, overlapping risks may be less affected by any single additional stressor, such that the marginal psychological impact of repeated victimization is attenuated or diluted among those already highly disadvantaged [42]. In contrast, cumulative disadvantage (or cumulative victimization) perspectives emphasize that victimization compounds existing marginalization, depleting already scarce coping resources and amplifying psychological distress with each additional exposure [43–45]. Both perspectives have empirical support, and distinguishing between them depends in part on the social contexts in which violence occurs.

Gang contexts may be particularly relevant to cumulative disadvantage dynamics. Violence in gang settings is often relational, retaliatory, and socially embedded, meaning that threats and victimization extend beyond the individual to their peers and associates [10, 46]. Prior victimization is a common precursor to gang involvement [12, 47], but gang membership also involves selection into environments where violence is expected, recurrent, and personally meaningful [48, 49]. Although gang affiliation may provide social identity, belonging, and perceived protection [50], these same ties can expand one’s sphere of vulnerability, heighten anticipatory stress, and undermine perceptions of safety and control [23, 27]. Under such conditions, repeated firearm exposure may compound psychological harm rather than produce emotional habituation. At the same time, existing data are limited, and it remains unclear whether gang involvement amplifies, attenuates, or does not meaningfully alter the psychological consequences of firearm victimization at the population level.

The present study addresses these gaps using data from a large, national survey of U.S. adults. We pursue two primary objectives. First, we examine whether respondents with a history of gang involvement report higher lifetime exposure to multiple forms of firearm victimization—including mass shootings, gun threats, and gunshot injury—than those without gang involvement, net of demographic, geographic, and socioeconomic factors. Second, among respondents who report firearm victimization, we assess whether self-reported psychological impacts differ by lifetime gang involvement. By providing national estimates of firearm victimization and associated mental health impacts, this study clarifies the extent to which firearm violence is concentrated within a potentially high-burden population and evaluates competing expectations regarding the psychological consequences of repeated exposure.

## Methods

### Sample and procedure

We fielded an online survey of U.S. adults (*n* = 10,000) from 16 to 30 January 2024 using YouGov, a major market research firm that recruits respondents through a large, actively maintained opt-in panel. YouGov employs a multistage, probability-matched sampling approach. A synthetic sampling frame, constructed using national benchmarks from the American Community Survey, was used to generate a target sample matched on age, gender, education, and race/ethnicity. Panelists who most closely match the target sample are selected using proprietary matching algorithms. Propensity score weights were generated using logistic regression models estimating inclusion in the matched sample based on age, education, gender, region, and race/ethnicity. Post-stratification weights were then applied using a four-way interlock of age, education, gender, and race/ethnicity, along with the 2020 presidential vote choice (including non-voters). All analyses apply weights to produce estimates designed to be representative of the U.S. adult population.

We targeted a large sample size to enhance population and relational inferences, particularly for low prevalence associations, behaviors, and experiences such as exposure to gun violence and gang involvement. Because this study used a pre-recruited opt-in panel rather than *de novo* probability sampling, a conventional response rate is not available. Panelists receive multiple survey invitations over time, and participation reflects panel engagement rather than study-specific recruitment. Survey invitations directed respondents to a study landing page describing “Exposure to Gun Violence in the United States.” Respondents provided digital informed consent before participation [51] and completed a 12-minute instrument consistent with American Association for Public Opinion Research (AAPOR) guidelines for online survey research (https://aapor.org/standards-and-ethics/best-practices/).

Eligibility criteria included being aged 18 years or older, residing in the United States, providing informed consent, and completing the survey with acceptable data quality. Cases were excluded for ineligibility, incomplete responses, quota fills, unsuccessful demographic matching, or poor data quality. Data quality screening included standard attention-check items, completion-time thresholds, and internal consistency checks. Participants received modest compensation through YouGov’s incentive system. To meet the target sample size within a short fielding window, approximately 2,400 completed surveys were obtained through a subcontracted panel vendor, a common practice in large national surveys [52]. Surveys obtained through the subcontracted panel followed identical recruitment, weighting, survey administration, and quality-control procedures; however, we were unable to assess whether there were differences between panels [53]. All de-identified data, code, and materials are archived via the Open Science Framework at https://osf.io/2rnby/.

## Measures

Full item wording and response options are provided in Appendix Table 5.

*Gun Violence Exposure.* Lifetime firearm victimization was measured with five dichotomous items adapted from prior national surveys and epidemiologic studies of gun violence exposure [4, 5, 31, 38], with wording designed to balance conceptual clarity and respondent comprehension. Respondents indicated whether they had ever experienced each of the following:

1. *Direct exposure to a mass shooting*, defined as being physically present during a public shooting in which ≥ 4 individuals were shot, consistent with combined Congressional Research Service and Gun Violence Archive definitions [30]. Physical presence required that the respondent either saw the shooter or bullets were fired toward them.
2. *Threatened with a firearm*, defined as being directly threatened at gunpoint.
3. *Shot at but not struck*, capturing incidents in which bullets were fired toward the respondent without physical injury.
4. *Shot*,* accidental*, defined as being unintentionally injured by gunfire.
5. *Shot*,* intentional*, defined as being deliberately shot by another person; this measure explicitly excludes self-inflicted firearm injuries or suicide attempts.

Each item was coded as 1 (“yes”) or 0 (“no”). For firearm victimization types that commonly recur (threatened with a firearm; shot at but not struck), respondents who endorsed lifetime exposure were also asked whether the experience occurred more than once, allowing assessment of repeated exposure.

*Mental Health Impacts.* For each firearm victimization type endorsed, respondents were asked whether they experienced specific psychological reactions following that event. Items assessed anxiety, fear, depression, panic attacks, post-traumatic stress symptoms, and changes in sleep, appetite, or concentration. These items captured whether symptoms occurred at any point following the firearm exposure rather than within a fixed recent lookback period (e.g., past month or past year).

Based on prior population mental health research [for a review, see 37], we constructed five dichotomous outcomes for each exposure: (1) any mental health impact (composite), (2) anxiety/fear (pooled), (3) depression-related symptoms (combining depression, sleep, appetite, or concentration changes), (4) panic attacks, and (5) post-traumatic stress symptoms. Outcomes were coded as 1 if the respondent endorsed the symptom and 0 otherwise. Although these measures do not represent clinical diagnoses, symptom-based self-report indicators are widely used in large-scale population surveys and have demonstrated validity for capturing common mental health problems [54–56].

*Gang Membership.* Gang involvement was measured using a validated self-nomination item: “Have you ever been a member of a gang on the street or in prison?” (yes = 1; no = 0). Self-nomination is widely regarded as a reliable single-item indicator of gang status across general population, school-based, neighborhood, and correctional samples [12, 35]. The item captures lifetime involvement rather than current or recent gang membership, and the inclusion of both street and prison contexts reflects the interconnected nature of U.S. gang networks.

*Covariates.* Covariates were selected a priori based on prior evidence linking them to both gang involvement and firearm victimization [4–12]. Demographic covariates included age (continuous), sex (male/female), and race/ethnicity. Sex was measured using a binary item and did not capture gender identity.

Geographic covariates included census region (West, South, Midwest, Northeast) and separate indicator variables for residence in California, Florida, New York, or Texas. State indicators were included alongside region to capture within-region heterogeneity in firearm exposure, policy environments, and population size. These indicators are mutually exclusive (e.g., respondents in California were coded independently from “West”).

Early-life contextual covariates included retrospective reports of parental education (years), growing up in a single-parent household, and whether gangs or frequent gunfire were present in the respondent’s neighborhood before age 18. Adult covariates included educational attainment (years), household income, and current firearm ownership. Firearm ownership reflects ownership at the time of the survey and does not capture lifetime ownership or access. The firearm ownership item did not distinguish between individual and household ownership, although respondents were asked separately if anyone else in their household owned any guns [57]. Missing covariate values for parental education and income were addressed using mean assignment with dummy variable adjustment.

## Analytic strategy

Analyses proceeded in four steps. First, we examined weighted descriptive statistics for demographic and socioeconomic characteristics, stratified by self-reported lifetime gang membership. Second, we compared the prevalence of firearm victimization across gang and non-gang adults. Third, we estimated multivariable logistic regression models predicting each form of gun violence exposure from gang membership, adjusting for all covariates. Adjusted odds ratios and predicted probabilities were calculated with covariates held at their mean values. Finally, for each type of firearm exposure, we compared the prevalence of mental health impacts between gang and non-gang respondents. All analyses were conducted using Stata 18.0 Standard Edition.

## Results

Table 1 presents weighted descriptive characteristics of the study sample, stratified by lifetime gang membership. Consistent with national averages, respondents had a mean age of 48.8 years and were evenly distributed by sex. 63% identified as White, 12% as Black, and 16% as Hispanic. Respondents resided across all U.S. census regions. Both parental and respondent educational attainment averaged nearly 14 years, consistent with completion of a two-year college degree. Roughly one-quarter of adults reported growing up in a single-parent household, in neighborhoods where gangs were present, or where they heard gunshots. Slightly more than one-quarter of respondents reported current firearm ownership.

**Table 1:** Descriptive statistics for the study sample by lifetime gang membership

	Full sample (*n* = 10,000) Mean/% (SD)	No lifetime gang (*n* = 9,759) Mean/% (SD)	Lifetime gang (*n* = 241) Mean/% (SD)
*Demographic measures*			
Age (in years)	48.75 (17.84)	48.98 (17.86)	39.61 (14.64)
Male	48.66%	47.96%	77.35%
White	62.78%	63.20%	45.86%
Black	12.46%	12.22%	22.13%
Hispanic	16.04%	15.86%	23.37%
Asian	3.04%	3.09%	1.15%
All other races and ethnicities^1^	5.67%	5.63%	7.49%
*Census/State region measures*			
West	12.53%	12.48%	14.90%
South	20.70%	20.93%	11.29%
Midwest	21.75%	21.74%	22.17%
Northeast	9.83%	9.80%	10.98%
California	11.04%	10.92%	15.73%
Florida	7.14%	7.17%	5.73%
New York	8.71%	7.79%	8.71%
Texas	10.49%	9.17%	10.49%
*Adolescent socioeconomic measures*			
Parental Education (in years)	13.82 (2.12)	13.83 (2.12)	13.57 (2.11)
One Parent Household	27.51%	26.81%	56.15%
Neighborhood Gang Activity	23.43%	22.01%	80.98%
Neighborhood Gunshots	23.75%	22.73%	65.20%
*Adulthood socioeconomic measures*			
Education (in years)	13.85 (2.29)	13.87 (2.29)	13.00 (2.06)
Income (in thousands)	73.28 (71.89)	73.62 (71.74)	59.77 (76.75)
Own a Firearm	27.09%	26.79%	39.48%

All values are weighted to be representative of adults in the United States. (SD) refers to standard deviation. ^1^All other races and ethnicities include respondents identifying as Native American, Middle Eastern, multiracial, or another race/ethnicity not listed separately

Overall, 2.4% of adults (95% confidence interval [CI]: 2.1–2.8%) reported lifetime gang membership. Compared with respondents without gang involvement, and consistent with prior research [12], those with gang involvement were younger, more likely to be male, and more likely to identify as Black or Hispanic. Gang-involved respondents were also more likely to report growing up in single-parent households and in neighborhoods where gangs and gunfire were present. In adulthood, they reported lower educational attainment and household income and higher rates of firearm ownership.

Table 2 presents weighted lifetime prevalence estimates for five forms of firearm victimization by gang membership. In the full sample, 7.0% (95% CI: 6.4–7.5%) reported ever being present at a mass shooting, 18.4% (95% CI: 17.5–19.2%) reported having been threatened with a firearm, 7.3% (95% CI: 6.7–7.8%) reported having been shot at but not struck, 2.3% (95% CI: 2.0–2.6%) reported an accidental gunshot injury, and 2.4% (95% CI: 2.1–2.8%) reported an intentional gunshot injury.

**Table 2:** Firearm victimization by lifetime gang and non-gang membership

	Full sample (*n* = 10,000)	No lifetime gang (*n* = 9,759)	Lifetime gang (*n* = 241)
Direct exposure to mass shooting	6.95%	6.11%	40.77%
Threatened with a firearm	18.37%	17.58%	50.41%
Shot at, but not struck	7.28%	6.60%	35.02%
Shot at, accidental	2.30%	1.98%	15.51%
Shot, intentional	2.43%	2.10%	15.58%

All values are weighted to be representative of adults in the United States

Prevalence estimates were higher among respondents with lifetime gang membership for all five firearm victimization measures. Among gang-involved respondents, 40.8% reported ever being present at a mass shooting, 50.4% reported having been threatened with a firearm, 35.0% reported having been shot at but not struck, 15.5% reported an accidental gunshot injury, and 15.6% reported an intentional gunshot injury. These differences correspond to odds ratios of 10.58 (95% CI: 7.73–14.47) for mass shooting exposure, 4.8 (95% CI: 3.52–6.45) for threatened with a firearm, 7.6 (95% CI: 5.48–10.64) for shot at, 9.1 (95% CI: 5.83–14.18) for accidental shooting, and 8.6 (95% CI: 5.60-13.12) for intentional shooting. Across all five indicators, gang membership was associated with at least 13-percentage point differences in firearm victimization. Table 3 presents adjusted odds ratios from multivariable logistic regression models estimating the association between lifetime gang membership and each firearm victimization outcome. All models adjusted for demographic characteristics, geographic indicators, early-life contextual variables, adult socioeconomic factors, and current firearm ownership (see Appendix Table 6). After adjustment, lifetime gang membership was associated with higher odds of all five firearm victimization outcomes. Adjusted odds ratios ranged from 2.33 (95% CI: 1.67–3.24) for being threatened with a firearm to 3.97 (95% CI: 2.75–5.73) for direct exposure to a mass shooting. Predicted probabilities derived from these models indicated higher estimated probabilities of firearm victimization among gang-involved respondents across all outcomes.

**Table 3:** Adjusted odds ratios and predicted probabilities in logistic regression models estimating associations of lifetime gang membership to firearm victimization

	Adjusted odds ratio	Confidence interval (95%)	*Pr* (Y = 1 | non-gang)	*Pr* (Y = 1 | gang)
Direct exposure to mass shooting	3.97	[2.75, 5.73]	0.033	0.121
Threatened with a firearm	2.33	[1.67, 3.24]	0.077	0.175
Shot at, but not struck	2.97	[1.96, 4.52]	0.048	0.130
Shot, accidental	2.94	[1.85, 4.69]	0.012	0.035
Shot, intentional	2.86	[1.77, 4.63]	0.014	0.039

All values are weighted to be representative of adults in the United States. Logistic regression models include demographic (age, sex, race/ethnicity), region/state (West, South, Midwest, Northeast, California, Florida, New York, and Texas), early-life (parent education, one-parent household, neighborhood gang activity, and neighborhood gunshots), and adult (education, income, and firearm ownership) covariates. Mean assignment with dummy variable adjustment was used for measures with missing information (parent education and income). Two-sided 95% confidence intervals are reported. Predicted probabilities, *Pr*, are based on holding all variables at mean values

Among respondents who reported lifetime exposure to firearm victimization types that commonly recur, repeated exposure was more frequently reported by gang-involved respondents (see Appendix Table 7). Among those who had ever been threatened with a firearm, 60.2% of respondents with gang involvement reported experiencing more than one such incident, compared with 42.6% of respondents without gang involvement. Among respondents who had been shot at but not struck, 60.0% of gang-involved respondents reported multiple incidents, compared with 39.7% of non-gang respondents. Repeated accidental or intentional gunshot injuries were less common in both groups. Among respondents reporting accidental gunshot injury, 33.2% of gang-involved respondents and 28.0% of non-gang respondents reported more than one incident. Among respondents reporting intentional gunshot injury, corresponding figures were 29.4% and 27.9%, respectively.

Cumulative exposure across distinct firearm victimization categories also differed by gang membership. Just 17% of gang-involved respondents reported no lifetime firearm victimization, compared with 75.0% of non-gang respondents. Among respondents reporting any exposure, the most common number of distinct firearm victimization types was one for non-gang respondents and two for gang-involved respondents. 38% of gang-involved respondents reported experiencing two distinct firearm victimization types, compared with 17.8% of non-gang respondents reporting at least one type.

Table 4 presents the weighted prevalence of reported mental health impacts following each firearm victimization type, stratified by gang membership. Across all exposure types, a majority of respondents reported at least one psychological impact following firearm victimization. Among respondents present at a mass shooting, 81.1% of non-gang respondents and 87.3% of gang-involved respondents reported at least one mental health impact. Among those threatened with a firearm, corresponding proportions were 65.9% and 73.0%. For respondents who had been shot at but not struck, 57.6% of non-gang respondents and 66.0% of gang-involved respondents reported at least one impact. Prevalence estimates for specific mental health outcomes—including anxiety or fear, depression-related symptoms, panic attacks, and post-traumatic stress symptoms—varied by exposure type and gang membership. Across the 25 comparisons of exposure type and mental health outcome, prevalence estimates were higher among gang-involved respondents in 20 comparisons.

**Table 4:** Prevalence of mental health impacts of firearm victimization by lifetime gang and Non-Gang membership

	No lifetime gang (*n* = 9,758)		Lifetime gang (*n* = 241)	
	f	%	f	%
**Direct exposure to mass shooting**	596	6.1%	98	40.8%
Mental health impact:				
Any type		81.1%		87.3%
Anxiety/fear		58.1%		51.6%
Depression		54.4%		60.4%
Panic attacks		27.1%		30.2%
Post-traumatic stress		25.2%		29.5%
**Threatened with a firearm**	1716	17.6%	121	50.4%
Mental health impact:				
Any type		65.9%		73.0%
Anxiety/fear		56.8%		51.7%
Depression		38.7%		55.9%
Panic attacks		21.1%		23.1%
Post-traumatic stress		24.5%		36.8%
**Shot at, but not struck**	644	6.6%	84	35.0%
Mental health impact:				
Any type		57.6%		66.0%
Anxiety/fear		47.5%		54.6%
Depression		34.1%		49.7%
Panic attacks		16.0%		26.0%
Post-traumatic stress		22.1%		28.8%
**Shot, accidental**	193	2.0%	37	15.5%
Mental health impact:				
Any type		71.4%		80.3%
Anxiety/fear		44.2%		33.3%
Depression		48.8%		55.5%
Panic attacks		27.3%		24.4%
Post-traumatic stress		22.1%		12.5%
**Shot, intentional**	206	2.1%	38	15.6%
Mental health impact:				
Any type		72.7%		75.9%
Anxiety/fear		51.5%		53.5%
Depression		49.1%		42.5%
Panic attacks		22.6%		28.8%
Post-traumatic stress		32.3%		36.1%

All values are weighted to be representative of adults in the United States

Adjusted logistic regression models comparing mental health impacts by gang membership among exposed respondents are reported in Appendix Table 8. In these models, relatively few differences by gang membership reached statistical significance, despite evidence of greater repeated exposure to gun violence. Statistically significant adjusted differences were observed for depression-related symptoms (OR: 2.32; 95% CI: 1.44–3.74) and post-traumatic stress symptoms (OR: 2.09; 95% CI: 1.26–3.49) following firearm threats; depression-related symptoms (OR: 2.03; 95% CI: 1.08–3.82) and panic attacks (OR: 2.28; 95% CI: 1.14–4.59) following being shot at but not struck; and anxiety or fear (OR: 0.36; 95% CI: 0.14–0.93) following accidental gunshot injury.

## Discussion

Using a national sample of U.S. adults, this study provides new evidence on the concentration of firearm victimization among individuals with a history of gang involvement and the psychological impacts associated with those experiences. Three core findings emerge. First, lifetime gang involvement was strongly associated with substantially higher exposure to every form of firearm victimization examined, including mass shooting presence, gun threats, and gunshot injury. Second, these associations persisted after adjustment for demographic, geographic, and socioeconomic factors, indicating that gang involvement captures relational and contextual risks not fully explained by socioeconomic disadvantage alone. Third, among adults who experienced firearm victimization, self-reported psychological impacts were common in both gang-involved and non-gang groups, with differences by gang status generally modest.

The magnitude and consistency of the exposure disparities reinforce long-standing criminological and public health research demonstrating that gang-involved individuals are disproportionately embedded in environments where serious violence is likely [12]. Prior longitudinal and network-based studies show that gang members occupy central positions in co-offending and victimization networks and face elevated risks of assault and gunshot injury relative to non-gang peers [10, 11, 22, 46]. The present findings extend this work by demonstrating that these patterns are evident at the national level and across multiple forms of firearm victimization. Even after accounting for early-life neighborhood violence, family structure, education, income, and firearm ownership, gang involvement remained associated with two- to four-fold higher odds of firearm exposure. This suggests that gang involvement reflects relational processes—such as retaliatory norms, contested space, and network proximity to violence—that are not reducible to individual or community characteristics.

Our prevalence estimates are broadly consistent with other national surveys, supporting the validity of our exposure measures. National polling shows that roughly one in five adults report being threatened with a gun, and 3–4% report having been shot, similar to the 18% threatened and 7% shot at in the present study [31]. Survey research with Black adults likewise documents high rates of direct firearm exposure [9]. Large-scale studies using Add Health data similarly identify nontrivial adult lifetime risks of being shot or shot at [58]. This convergence across independent samples underscores both the credibility of our measures and the exceptionally high exposure levels among respondents with a history of gang involvement.

The prevalence of mass shooting exposure among respondents involved in gangs in their lifetime merits particular attention. Whereas a small minority (6%) of non-gang adults reported ever being present at a mass shooting, a large share of adults with gang involvement (41%) did so. Because the measure used here did not exclude incidents labeled as “gang-related,” these results highlight substantial overlap between categories of firearm violence that are often treated as analytically distinct [32]. For individuals embedded in chronically violent social environments, mass shootings may represent an extension of routine risk rather than an isolated or exceptional event [53]. This finding has implications for firearm violence surveillance and prevention efforts that focus narrowly on high-profile incidents while overlooking the populations most frequently exposed to gunfire.

Firearm victimization was associated with high levels of reported psychological impact across all exposure types and among both gang-involved and non-gang adults. Consistent with prior research [37–41], mass shooting exposure was associated with particularly high prevalence of reported distress, while being shot at without injury was associated with comparatively lower—but still substantial—levels. Still, the findings affirm that firearm victimization contributes to population-level psychological morbidity, not only in large, high-profile events but also in more routine interpersonal encounters [59, 60]. Contrary to expectations that chronic exposure might blunt emotional responses, gang-involved adults did not report systematically lower levels of psychological impact. Across most comparisons, prevalence estimates were similar or higher among gang-involved victims, although relatively few differences reached statistical significance in adjusted models. Within the limitations of the study (see below), this pattern is more consistent with cumulative disadvantage than with disadvantage saturation [42]: repeated exposure to firearm violence among gang-involved individuals does not appear to eliminate psychological distress and may instead contribute to persistent vulnerability. At the same time, the data do not permit definitive tests of competing theoretical mechanisms, and the results should be viewed as suggestive rather than conclusive.

These findings have several implications for clinical practice and violence prevention. First, they underscore the importance of routinely assessing firearm victimization, including threats and near-miss events, in clinical, correctional, and behavioral health settings. Nonfatal exposures are common, particularly among individuals with gang involvement, and are associated with substantial psychological distress. Brief, validated self-report measures commonly used in population mental health surveillance [54, 56]—including the WHO World Mental Health Surveys [55] and global trauma exposure studies [61]—can support early identification of trauma-related symptoms. Gang-involved individuals may require continued follow-up and accessible trauma-focused services not only in healthcare settings but also in schools, community mental health centers, and other neighborhood institutions that often serve as first points of contact for affected individuals [62].

Second, violence-intervention efforts should more fully integrate trauma-informed components. Hospital-based violence intervention programs, credible messenger models, and community violence intervention programs increasingly serve gang members with significant victimization histories [63–67], yet many focus on service linkage and conflict mediation with less emphasis on mental health [68]. For gang-involved adults, access to evidence-based treatments—including trauma-focused cognitive-behavioral therapy, culturally responsive counseling, and appropriate pharmacotherapy—is essential [69]. Evaluations of HVIPs and CVI programs should explicitly measure mental health outcomes to determine whether trauma-informed enhancements improve both psychological and violence-related endpoints [70].

Third, policy efforts that expand access to affordable, culturally responsive mental health services in high-violence neighborhoods may mitigate long-term consequences of firearm victimization [71]. Current national discourse around “group” or “community violence” and CVI strategies has shifted away from explicitly naming gangs [72], partly to avoid racialized or overly broad labels [73]. However, this broader framing risks obscuring the acute and disproportionate risks faced by individuals with gang involvement. Our results reinforce that gang membership remains an independent correlate of firearm victimization. Following longstanding patterns of local “denial” about gang problems in communities and police agencies [74, 75], failing to acknowledge gangs explicitly risks rendering these high-risk relational environments less visible in prevention, clinical assessment, and service delivery. Effective policy requires attention to both structural conditions and the relational environments in which violence is embedded, alongside investments in upstream strategies such as neighborhood development, economic opportunity, and youth engagement [76].

This study has limitations. The cross-sectional design precludes causal inference and does not allow determination of temporal ordering between gang involvement, firearm victimization, and mental health symptoms. All key exposure measures (i.e., gang membership and firearm victimization) capture lifetime experiences, while mental health impacts reflect retrospective reports of symptoms following those events rather than current or recent diagnoses. Symptoms may have resolved, recurred, or been influenced by subsequent experiences, which likely attenuates observed differences in mental health outcomes by gang status.

Second, all measures were self-reported and may be subject to recall error or reporting biases. Our measure of being “shot at but not struck” does not distinguish between intentional targeting and accidental or stray gunfire, which may introduce heterogeneity in psychological responses. Similarly, the firearm ownership item does not differentiate individual from household ownership and may reflect access rather than ownership [57].

Third, the gang membership measure captures lifetime involvement but does not assess duration, intensity, timing, or type of gang involvement, all of which may shape exposure and psychological outcomes [33]. Unmeasured confounding is also possible. Early adverse childhood experiences and other forms of trauma not captured in this study may be associated with both gang involvement and firearm victimization, potentially biasing estimates.

Fourth, the sample was recruited through an online opt-in panel and excludes individuals without reliable internet access, which may limit representativeness for some highly marginalized populations. Although post-stratification weighting improves demographic representativeness, differential participation by prior exposure to gun violence cannot be ruled out.

Fifth, while the overall sample is large, relatively few participants reported gang membership or specific victimization types, limiting statistical power for subgroup analyses and increasing uncertainty around some estimates (see Appendices). Future work should incorporate richer measures of group involvement, social networks, and longitudinal trajectories, and mixed-methods approaches may clarify how gang-involved individuals interpret and cope with firearm violence.

Finally, these findings arise from a U.S. context with high firearm availability and gun violence [77]. Although underlying mechanisms may apply elsewhere, replication across diverse settings is necessary to understand how different regulatory environments and gang structures shape patterns of exposure and psychological response. Research from Central America, the Caribbean, and parts of Europe similarly documents the concentration of violence within gang networks, suggesting that the dynamics observed here may have broader relevance even if absolute rates differ [12].

## Conclusion

Using a national sample of U.S. adults, this study demonstrates that individuals with a history of gang involvement experience substantially higher lifetime exposure to multiple forms of firearm victimization than those without such involvement. These disparities persist after accounting for demographic, geographic, and socioeconomic characteristics, indicating that gang involvement captures relational and contextual risks that are not fully explained by measured disadvantage alone.

Across all forms of firearm victimization examined, self-reported psychological impacts were common among both gang-involved and non-gang adults. Differences in mental health outcomes by gang status were generally modest, but there was no evidence that repeated exposure among gang-involved individuals eliminated psychological distress. Within the limits of lifetime and retrospective measurement, the findings are more consistent with cumulative disadvantage—characterized by the accumulation of vulnerability—than with disadvantage saturation or emotional desensitization.

Taken together, these results underscore the concentration of firearm violence exposure within gang-involved populations and the substantial psychological burden associated with that exposure. Public health and violence-prevention efforts that focus narrowly on high-profile incidents or on structural risk factors alone may miss the relational environments in which firearm violence is most frequent and consequential. Recognizing gang-involved individuals as both highly exposed to firearm violence and vulnerable to its psychological effects is essential for effective clinical assessment, trauma-informed intervention, and comprehensive firearm-injury prevention strategies.

## Data Availability

All data and materials used in the analysis, including the survey instrument, are archived via the Open Science Framework project at [https://osf.io/2rnby/](https:/osf.io/2rnby) .

## Appendix

See Tables 5, 6, 7 and 8.

**Table 5:** Item wording and response categories

Variable	Item wording	Response categories
Age	In what year were you born? In what month were you born?	Open numeric response
Sex (Male)	Are you male or female?	Select one
Race/Ethnicity	What racial or ethnic group best describes you?	White; Black; Hispanic; Asian; Native American; Middle Eastern; Two or more races; Other; Don’t Know
Region/State	Respondent report of zip code, county, and state	Zip Codes; Counties; and States (recoded into 4 states and 4 census regions)
Parent education	What is the highest level of education *your mom* achieved? What is the highest level of education *your dad* achieved?	Less than 8th grade; More than 8th grade but less than high school diploma; High school diploma; GED; 2 year degree; 4 year degree; Postgraduate degree; Don’t Know (converted to years of education, averaged across parent(s), and standardized)
One-Parent Household	Did you grow up in a household where only one parent was present?	Yes (coded 1); No (coded 0)
Neighborhood gang activity (growing up)	Did you grow up in a neighborhood where gangs were present?	Yes (coded 1); No and Don’t know (coded 0)
Neighborhood gunshots (growing up)	How often did you hear gun shots in your neighborhood growing up?	Never and Don’t Know (coded 0); Almost Never, Somewhat Often, and Often (coded 1)
Education (adult)	What is the highest level of education you have completed?	No High school; High school graduate; Some college; 2-year; 4-year; Post-grad (converted to years of education and standardized)
Income	Thinking back of the last year, what was your family’s annual income?	Less than $10,000; 10-19.9k; 20-29.9k; 30-39.9k; 40-49.9k; 50-59.9k; 60-69.9k; 70-79.9k; 80-99.9k; 100-119.9k; 120-149.9k; 150-199.9k; 200-249.9k; 250-349.9k; 350-499.9k; 500k or more; (converted to mid-point and standardized)
Own a firearm	How many guns (NOT including air guns, such as paintball, BB or pellet guns) do you own?	Own one firearm, own 2–5 firearms, own 6–9, and own 10 or more (coded 1); own none (coded 0)
Lifetime gang membership	Have you ever been a member of a gang on the street or in prison?	Yes (1); No (0)
Direct exposure to mass shooting	Have you personally ever been physically present on the scene of a mass shooting in your lifetime? By physically present we mean bullets were fired in your direction, you could see the shooter, or hear gunfire.	Yes (1); No (0); Don’t know
Firearm victimization	Which of the following have happened to you? (Select all that apply.)	Witnessed injury; Threatened with gun; Shot accidentally; Shot intentionally; Shot at; None
Repeat firearm exposure	You mentioned experiencing [prior event]. How many times has this occurred in your lifetime?	Open numeric response
Mental health impacts	After the incident, did you experience any of the following? (Select all that apply.)	Anxiety; Depression; PTSD symptoms; Panic attacks; Sleep difficulty; Eating changes; Fear; Concentration problems; None

**Table 6:** Descriptive statistics for repeated exposure to types of gun victimization

	Full sample (*n* = 10,000)		No lifetime gang (*n* = 9,759)		Lifetime gang (*n* = 241)	
	Any	> 1	Any	> 1	Any	> 1
Threatened with a firearm	18.4%	43.7%	17.6%	42.6%	50.4%	60.2%
Shot at, but not struck	7.3%	42.0%	6.6%	39.7%	35.0%	60.0%
Shot, accidental	2.3%	28.8%	2.0%	28.0%	15.5%	33.2%
Shot, intentional	2.4%	28.4%	2.1%	27.9%	15.6%	29.4%
Unique exposure count						
0	73.7%		75.0%		17.4%	
1	18.1%		17.8%		28.9%	
2	6.0%		5.2%		38.5%	
3	1.9%		1.7%		10.6%	
4	28.0%		0.2%		3.7%	
5	9.0%		0.1%		1.0%	

All values are weighted to be representative of adults in the United States

**Table 7:** Multivariable logistic regression models estimating associations of lifetime gang membership to firearm victimization

	Direct exposure to mass shooting		Threatened with a firearm		Shot at, but not struck		Shot, accidental		Shot, intentional	
	AOR	95% CI	AOR	95% CI	AOR	95% CI	AOR	95% CI	AOR	95% CI
Lifetime gang membership	**3.97**	**[2.75**,**5.73]**	**2.33**	**[1.67**,**3.24]**	**2.97**	**[1.96**,**4.52]**	**2.94**	**[1.85**,**4.69]**	**2.86**	**[1.77**,**4.63]**
Demographic										
Age (in years)	**0.51**	**[0.45**,**0.58]**	1.05	[0.99,1.12]	1.06	[0.97,1.16]	**0.72**	**[0.60**,**0.87]**	0.88	[0.73,1.04]
Male^a^	**1.35**	**[1.11**,**1.63]**	**1.34**	**[1.19**,**1.50]**	**2.42**	**[2.02**,**2.90]**	**1.60**	**[1.17**,**2.17]**	**2.17**	**[1.56**,**3.02]**
Black^b^	1.09	[0.85,1.40]	0.85	[0.72,1.01]	0.92	[0.72,1.18]	1.07	[0.72,1.60]	1.52	[1.03,2.26]
Hispanic^b^	**0.71**	**[0.52**,**0.97]**	0.83	[0.67,1.02]	**0.68**	**[0.49**,**0.93]**	0.77	[0.47,1.26]	1.05	[0.66,1.67]
Asian^b^	**0.44**	**[0.24**,**0.81]**	**0.58**	**[0.38**,**0.86]**	**0.34**	**[0.15**,**0.78]**	0.49	[0.15,1.63]	1.58	[0.72,3.48]
Other^b^	0.80	[0.55,1.16]	1.42	[1.16,1.76]	1.29	[0.96,1.75]	1.09	[0.64,1.84]	1.35	[0.79,2.30]
Census/State Region										
South^c^	0.90	[0.63,1.28]	0.92	[0.75,1.13]	**0.68**	**[0.51**,**0.91]**	0.74	[0.41,1.32]	0.76	[0.42,1.37]
Midwest^c^	0.82	[0.58,1.16]	0.88	[0.72,1.08]	0.80	[0.60,1.06]	0.79	[0.45,1.37]	0.98	[0.55,1.74]
Northeast^c^	0.79	[0.52,1.20]	**0.65**	**[0.51**,**0.84]**	**0.57**	**[0.39**,**0.84]**	0.51	[0.26,1.02]	0.58	[0.29,1.16]
California^c^	**1.56**	**[1.08**,**2.24]**	**0.76**	**[0.59**,**0.98]**	0.74	[0.51,1.08]	0.94	[0.52,1.71]	1.38	[0.76,2.52]
Florida^c^	0.96	[0.59,1.57]	0.76	[0.58,1.01]	**0.62**	**[0.42**,**0.92]**	0.93	[0.43,2.01]	0.90	[0.41,1.97]
New York^c^	**1.72**	**[1.17**,**2.51]**	**0.71**	**[0.54**,**0.94]**	**0.42**	**[0.26**,**0.68]**	1.28	[0.70,2.34]	1.45	[0.78,2.67]
Texas^c^	1.06	[0.70,1.60]	**0.70**	**[0.54**,**0.91]**	**0.68**	**[0.46**,**1.00]**	1.23	[0.66,2.30]	1.08	[0.57,2.05]
Early-life measures										
Parental education (in years)^d^	**1.17**	**[1.05**,**1.30]**	0.97	[0.90,1.03]	1.00	[0.90,1.11]	**1.28**	**[1.05**,**1.56]**	1.12	[0.93,1.34]
One Parent Household	**1.91**	**[1.57**,**2.34]**	**1.33**	**[1.17**,**1.51]**	**1.45**	**[1.19**,**1.76]**	**2.27**	**[1.65**,**3.12]**	**1.86**	**[1.35**,**2.58]**
Neighborhood Gang Activity	**2.08**	**[1.65**,**2.62]**	**1.80**	**[1.55**,**2.09]**	**1.66**	**[1.33**,**2.06]**	**1.42**	[0.96,2.11]	1.46	[0.99,2.15]
Neighborhood Gunshots	**2.94**	**[2.36**,**3.65]**	**1.85**	**[1.60**,**2.14]**	**2.48**	**[2.02**,**3.04]**	**2.94**	**[1.97**,**4.37]**	**2.68**	**[1.87**,**3.85]**
Adult Measures										
Education (in years)^d^	**1.20**	**[1.07**,**1.34]**	0.89	[0.83,0.95]	0.87	[0.77,0.97]	0.98	[0.81,1.18]	0.90	[0.75,1.09]
Income (in thousands)^d^	**1.15**	**[1.06**,**1.25]**	0.97	[0.90,1.05]	0.84	[0.74,0.95]	1.11	[0.98,1.26]	1.04	[0.90,1.20]
Own a Firearm	**1.51**	**[1.22**,**1.88]**	**1.38**	**[1.22**,**1.57]**	**2.02**	**[1.68**,**2.44]**	**1.89**	**[1.38**,**2.57]**	**1.58**	**[1.14**,**2.20]**
Constant	0.02	[0.01,0.02]	0.14	[0.12,0.17]	0.03	[0.02,0.04]	0.01	[0.00,0.01]	0.01	[0.00,0.01]

All values are weighted to be representative of adults in the United States. Mean assignment with dummy variable adjustment was used for measures with missing information (parent education and income). Bolded values indicate odds ratios where two-sided 95% confidence intervals do not overlap with one. ^a^Female is the reference category. ^b^White is the reference category. ^c^West is the reference category. ^d^ Standardized

**Table 8:** Adjusted odds ratios and predicted probabilities in logistic regression models estimating associations of lifetime gang membership to mental health impacts by firearm victimization types

	Odds ratio	95% CI	Pr (Y = 1| non-gang)	Pr (Y = 1| gang)
Direct exposure to mass shooting				
Any type	1.36	[0.66, 2.79]	0.85	0.89
Anxiety/fear	0.75	[0.44, 1.29]	0.59	0.52
Depression	0.99	[0.58, 1.67]	0.56	0.55
Panic attacks	0.99	[0.57, 1.73]	0.26	0.26
Post-traumatic stress	1.23	[0.68, 2.19]	0.24	0.28
Threatened with a firearm				
Any type	1.52	[0.92, 2.52]	0.69	0.77
Anxiety/fear	0.88	[0.54, 1.44]	0.58	0.55
Depression	**2.32**	**[1.44**,** 3.74]**	**0.37**	**0.58**
Panic attacks	1.22	[0.70, 2.12]	0.19	0.22
Post-traumatic stress	**2.09**	**[1.26**,** 3.49]**	**0.22**	**0.37**
Shot At, But Not Struck				
Any type	1.21	[0.65, 2.26]	0.59	0.64
Anxiety/fear	1.25	[0.67, 2.30]	0.48	0.53
Depression	**2.03**	**[1.08**,** 3.82]**	**0.32**	**0.49**
Panic attacks	**2.28**	**[1.14**,** 4.59]**	**0.12**	**0.24**
Post-traumatic stress	1.54	[0.83, 2.85]	0.20	0.27
Shot At, Accidental				
Any type	1.18	[0.34, 4.09]	0.80	0.83
Anxiety/fear	**0.36**	**[0.14**,** 0.93]**	**0.45**	**0.23**
Depression	0.94	[0.36, 2.41]	0.49	0.47
Panic attacks	0.55	[0.17, 1.74]	0.25	0.15
Post-traumatic stress	0.46	[0.12, 1.75]	0.18	0.09
Shot, Intentional				
Any type	0.83	[0.33, 2.14]	0.79	0.76
Anxiety/fear	0.87	[0.36, 2.14]	0.52	0.49
Depression	0.52	[0.22, 1.24]	0.50	0.35
Panic attacks	1.93	[0.75, 4.95]	0.18	0.30
Post-traumatic stress	1.37	[0.56, 3.35]	0.29	0.36

All values are weighted to be representative of U.S. adults. Logistic regression models include demographic (age, sex, race/ethnicity), region/state (West, South, Midwest, Northeast, California, Florida, New York, and Texas), early-life (parent education, one-parent household, gang activity, and gunshots in neighborhood), and adult (education, income, and firearm ownership) covariates. Mean assignment with dummy variable adjustment was used for missing information on parent education and income. Predicted probabilities, *Pr*, are based on holding all variables at mean values. Bolded values indicate odds ratios where two-sided 95% confidence intervals do not overlap with one
